# Comparison of Total Antioxidant Capacity in COPD, Asthma, and Asthma–COPD Overlap Patients

**DOI:** 10.3390/medicina61081340

**Published:** 2025-07-24

**Authors:** Melike Yüksel Yavuz, Muzaffer Onur Turan, Hayat Özkanay, Mehmet Köseoğlu

**Affiliations:** 1 Pulmonology Department, School of Medicine, İzmir Katip Çelebi University, 35620 İzmir, Türkiye; onurtura@yahoo.com; 2 Medical Biochemistry Department, School of Medicine, İzmir Katip Çelebi University, 35620 İzmir, Türkiye; hayatozkanay@hotmail.com (H.Ö.); mkoseoglu@yahoo.com (M.K.)

**Keywords:** antioxidant, COPD, asthma, asthma–COPD overlap, total antioxidant capacity

## Abstract

*Background and Objectives:* Asthma, COPD, and asthma–COPD overlap are obstructive lung diseases with inflammation at their core. Oxidative stress and impaired antioxidant balance play a significant role in etiopathogenesis. This study aimed to determine whether there are differences in total antioxidant capacity (TAC) between asthma, COPD, and asthma–COPD overlap. *Materials and Methods:* A total of 76 patients participated in this prospective cross-sectional study. TAC levels in fasting venous blood samples were measured using a biochemistry analyzer and the total antioxidant activity method (Architect C1600, Abbott Laboratories, IL, USA). *Results*: TAC levels were lower in COPD patients compared to asthma and ACO patients (*p* = 0.049 and 0.026, respectively). TAC levels were lower in current and former smokers compared to never smokers (*p* = 0.033). There was no significant correlation between TAC level and eosinophil count (*p* = 0.597) and FEV1 and FEV1/FVC (*p* = 0.372 and *p* = 0.189). *Conclusions*: Our results suggest that TAC levels may serve as a marker to differentiate COPD from asthma or ACO.

## 1. Introduction

Oxidative stress in the lungs occurs when the antioxidant capacity is overloaded or depleted due to infections, altered oxygen tension, systemic diseases, or environmental pollution. Pulmonary responses to oxidative stress include the activation of oxidases, lipid peroxidation, increases in nitric oxide level, and autophagy. These internal and external exposures and subsequent pulmonary responses contribute directly to the development of oxidative-stress-related diseases [1]. In a healthy lung, the integrity of the basement membrane reflects the balance of proteases and antiproteases. These enzymes are continuously synthesized by inflammatory cells (such as monocytes/macrophages, neutrophils, and eosinophils) and mesenchymal cells (such as fibroblasts, macrophages, endothelial cells, and epithelial cells). Patients with respiratory diseases such as chronic obstructive pulmonary disease (COPD), asthma, interstitial lung diseases, and cystic fibrosis experience high levels of reactive oxygen species (ROS) production and oxidative stress [2]. Although the lungs have strong enzymatic and non-enzymatic antioxidant defenses (GSH, ascorbic acid, uric acid, and vitamin E), the production of ROS, for example, due to continuous exposure to environmental pollutants, impairs lung antioxidant defenses and causes oxidative damage [3].

COPD is both a preventable and treatable disease that ranks in the top three for the number of deaths caused worldwide. The health burden of COPD is expected to increase in the coming decades as the aging population continues to be exposed to risk factors, particularly tobacco products [4]. Oxidative stress is increased in non-smokers, ex-smokers, and current smokers. At the same time, this inflammatory effect is increased with decreasing antioxidant capacity. Oxidative stress may be endogenous and/or exogenous. Endogenously, inflammatory cells, such as neutrophils, macrophages, fibroblasts, and epithelial cells, produce ROS [5]. Exogenous factors include smoking, environmental and/or occupational vapor–dust–gas–fume (VDGF) exposure, and air pollution. Although oxidative stress is thought to be the mechanism responsible for many pathophysiological changes in COPD, treatment strategies have not yet been developed [6].

As a result of complex underlying pathophysiological mechanisms, COPD patients experience persistent oxidative stress, increased levels of proinflammatory cytokines, increased numbers of CD4 and CD8 cells, elevated protease levels, and increased apoptosis and aging [3]. Biomarkers of oxidative stress are increased in the breath, systemic circulation, and sputum of COPD patients. Oxidative stress increases even more during exacerbations. In the GOLD 2024 report [4], it was recommended that patients be fed a diet rich in antioxidants. Among antioxidant agents, N-acetylcysteine, carbocysteine, and erdosteine are reported to reduce exacerbations.

Asthma is a heterogeneous disease characterized by chronic airway inflammation, with symptoms such as wheezing, shortness of breath, chest tightness, and coughing, varying in duration and severity. Asthma is a common disease affecting 1–29 percent of the population. Increased extracellular matrix protein production and an increase in proinflammatory cytokine levels in the airways, due to increased ROS production, are involved in its pathogenesis. Therefore, as stated in the GINA 2024 guidelines, a diet rich in antioxidants is recommended for asthma patients [7]. As in COPD, endogenous and exogenous oxidative stress occur in asthma. In asthma, the trigger initiates IgE production, the degranulation of mast cells, and the infiltration of eosinophils into the airways. Th2 cytokines (IL-13, IL-4, IL-5, and IL-9), Th1 cytokines (IFN-g, IL-12), and Th17 cytokines (IL-23) cause airway epithelial damage, characterized by high neutrophilic inflammation. ROS increases sensitization to allergens and increases Th2/Th1 cytokine secretion [3].

ROS are involved in the pathogenesis of chronic airway inflammation in asthmatic patients. The overproduction of ROS leads to increased malonic dialdehyde concentrations and total oxidant content. In addition, decreased antioxidant enzyme activity (superoxide dismutase, catalase, and glutathione peroxidase) in asthmatic patients also contributes to this pathway [8]. Although asthma–COPD overlap (ACO) is no longer favored, there is a group of patients in whom asthma and COPD can coexist, with common treatable features and clinical characteristics (e.g., eosinophilia, some degree of reversibility) [4]. In an animal study comparing the inflammatory profile, oxidative stress, and lung tissue remodeling in an ACO model, it was shown that lung function was altered, remodeling was induced, and inflammation and oxidative stress increased [9]. The etiopathogenesis of many chronic diseases lies in the lack of ROS neutralization as a result of the depletion of the antioxidant reserve system by the excessive accumulation of free radicals [10].

Antioxidant defenses regularly respond to the physiological production of oxidants. In this way, redox homeostasis is stabilized and protection against oxidative stress damage is provided. The total antioxidant capacity (TAC) is a marker that represents the scavenging of free radicals by a test solution or suspension. TAC measurement is of great importance in determining the body’s oxidant–antioxidant balance. TAC levels remain one of the most widely used methods for measuring the possible oxidant buffering capacity of a sample [11]. This study aimed to compare serum TAC levels in stable COPD, asthma, and ACO patients and to evaluate antioxidant activity in these diseases.

## 2. Materials and Methods

### 2.1. General Data

A total of 76 patients with stable COPD, asthma, and ACO who were admitted to the Chest Diseases Outpatient Clinic of İzmir Katip Çelebi University Atatürk Training and Research Hospital between August and December 2018 were included in this cross-sectional study. G*Power software (version 3.1.9.7; Heinrich Heine University Düsseldorf, Düsseldorf, Germany) was used to determine the sample size for our trial. The effect size of 0.35 with 80% power revealed that a total sample size of 82 was required to detect minimal clinically important differences at a 5% significance level. The trial ended after 82 patients with full records and laboratory tests were reached. Finally, the sample size was reduced to 76 after adopting the inclusion/exclusion criteria in detail. The definitive analysis excluded 3 patients due to outlier levels, while the blood samples of 3 patients were not appropriate for laboratory work. The exclusion criteria included patients who were pregnant, under 18 years old, and who had heart disease, kidney disease, cancer, or a history of surgery in the last month. A pulmonologist confirmed asthma, COPD, and ACO diagnoses according to the current GINA and GOLD reports for 2018. ACO is characterized by persistent airflow limitation, with some features usually associated with asthma and some features usually associated with COPD. Since there are no strictly accepted diagnostic criteria, patients with both asthma and COPD were included in this group in our study [12,13]. Eosinophil counts from the patients’ current complete blood counts were recorded. The forced expiratory volume in 1 second (FEV1), forced expiratory capacity (FVC), and FEV1/FVC values, based on the simple spirometry test (the Easy-On^®^ is a powerful PC-based ultrasonic diagnostic spirometer) in the last 6 months, were obtained from the patients’ records. The term current smoker was used for those who had smoked at least 100 cigarettes and were current smokers, former smoker was used for those who had smoked at least 100 cigarettes but had stopped smoking at the time of the interview, and never smoker was used for those who had never smoked or smoked fewer than 100 cigarettes in their lifetime [14].

### 2.2. Total Antioxidant Capacity Assessment

Venous blood samples taken from the patients in the morning while fasting were centrifuged at 4000 rpm for 10 min at +4 °C, and the serum samples to be separated were transferred to Eppendorf tubes. The prepared serum was stored in a deep freezer at −80 °C until the day of analysis, and all patients were studied together after collection. The TAC levels of the study patients were measured by means of the total antioxidant activity method in a biochemistry analyzer (Architect C1600, Abbott Laboratories, IL, USA) [15]. In this measurement method, the total antioxidant level of the sample, which is related to the absorbance change at 660 nm, was evaluated based on the conversion of the dark blue-green-colored 2,2′-azino-bis (3-ethylbenzothiazoline-6-sulphonic acid) radical cation (ABTS*+) to the colorless reduced ABTS form [16]. (±)-6-Hydroxy-2,5,7,8-tetramethylchromane-2-carboxylic acid (Trolox Equivalent), a vitamin E analog, was used for calibration. Two reagents were used for measurement: 0.4 M pH 5.8 acetate buffer was prepared for Reagent 1, and 30 mM pH 3.6 acetate buffer was prepared for Reagent 2. Then, a 2 mmol/L H_2_O_2_ solution was prepared using this buffer. Reagent 2 with a final ABTS concentration of 10 mmol/L was prepared using the last solution. The results were expressed as mmol Trolox Equiv./L.

### 2.3. Ethical Approval

Informed written consent was obtained from all participants. This study was structured prospectively, and it was performed in accordance with the Declaration of Helsinki and its amendments. Ethics committee approval was obtained from Izmir Katip Çelebi University Non-Drug Clinical Research Ethics Committee on 5 July 2018 with reference number 83.

### 2.4. Statistical Analysis

Statistical analyses were performed using the Statistical Package for the Social Sciences (SPSS) version 27.0 (IBM Corp., Armonk, NY, USA). Continuous variables are presented as mean ± SD if the data are normally distributed and median [min-max] if it is not normally distributed. Categorical variables are presented as numbers and percentages (n (%)). The normal distribution of continuous variables was investigated by normality tests. Pearson’s chi-square test was used to investigate the impact of categorical variables, while independent-sample *t*-tests and one-way ANOVA were used for continuous variables. Pearson correlational analysis was used to measure the strength and direction of the linear relationship between two continuous variables. The level of statistical significance was considered as *p* < 0.05.

## 3. Results

### 3.1. Baseline Characteristics of the Groups

Of the 76 patients, 61.8% (47) were male and 38.2% (29) were female. The study included 26 asthma, 30 COPD, and 20 ACO patients. The mean age of the participants was 55.6 ± 14.4. The smoking status of the participants was as follows: 19.7% (15) were never smokers, 28.9% (22) were former smokers, and 51.3% (39) were current smokers. Demographic data, spirometry values, and TAC levels of the patients are given in Table 1.

### 3.2. Comparison of TAC Levels of Groups

The mean TAC level of all participants was 1079.1 ± 261.4 mmol/L. The mean TAC levels were 1035.6 ± 245.5 mmol/L for males and 1149.6 ± 275.2 mmol/L for females. No statistically significant difference was found between male and female participants regarding TAC level (*p* = 0.065). The TAC level was 1167.9 ± 254.8 in never smokers, 1091.7 ± 320.9 in former smokers, and 1076.3 ± 261.4 mmol/L in current smokers. There was no statistical difference in TAC level according to smoking status (*p* = 0.960). When post hoc analyses were considered, a statistically significant decrease in TAC level was observed in the current and former smoker groups compared to never smokers (*p* = 0.033). The TAC level was 1154.8 ± 310.1 in the asthma patients, 1027.9 ± 203.6 in the COPD patients, and 1042.4 ± 260.9 mmol/L in the ACO patients. There was no statistically significant difference in TAC levels between the asthma, COPD, and ACO groups (*p* = 0.192). When post hoc analyses were considered, there was no statistically significant difference between the asthma and ACO groups regarding TAC level (*p* = 0.551). TAC levels were significantly lower in COPD patients compared to asthma and ACO patients (*p* = 0.049 and 0.026, respectively) (Table 2).

There was no correlation between FEV1 level and FEV1/FVC and TAC levels in patients (*p* = 0.372 and *p* = 0.189). When correlational analyses were performed between FEV1 levels and TAC values for asthma, COPD, and ACO patients, no significant relationship was found (*p* = 0.079, *p* = 0.132, *p* = 0.784, respectively). When correlational analyses were performed between FEV1/FVC and TAC values for asthma, COPD, and ACO patients, no significant relationship was found (*p* = 0.995, *p* = 0.986, *p* = 0.471, respectively) (Table 3).

**Figure 1:**
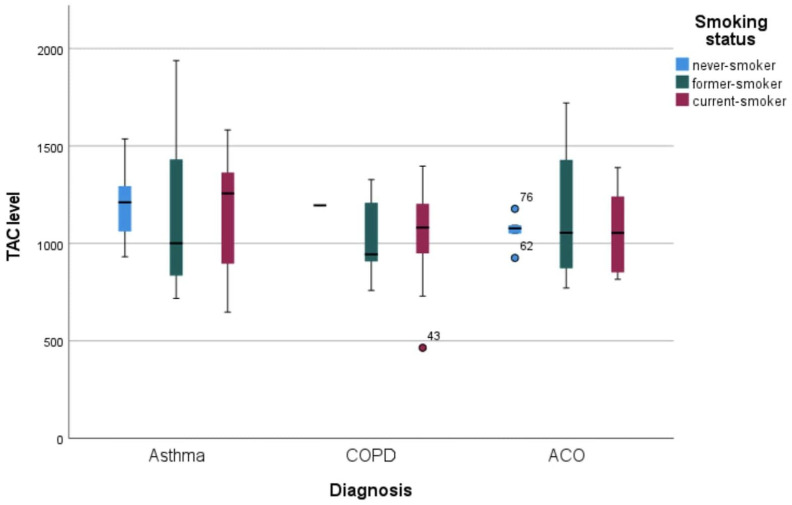
TAC levels of asthma, COPD, and ACO patients according to smoking status.

In never smokers, no significant difference was found in terms of TAC level according to the diagnosis of asthma and ACO (*p* = 0.710). There was no significant difference between former smokers and current smokers in terms of TAC levels according to the diagnosis status of asthma, COPD, and ACO (*p* = 0.754 and *p* = 0.546). The TAC levels of asthma, COPD, and ACO patients according to smoking status are shown in Figure 1. Mean whole blood eosinophil counts were 246.6 ± 172.1 in asthmatic patients, 185.6 ± 122.2 in COPD patients, and 366 ± 330 in ACO patients.

There was a statistically significant difference between the groups in terms of eosinophil counts (*p* = 0.016). However, in post hoc analyses, it could not be determined which groups were different. Observationally, we can say that eosinophil counts decrease in ACO, asthma, and COPD patients in this order. No significant correlation was found between eosinophil counts and TAC levels in all patients (*p* = 0.597). When correlational analyses were performed between eosinophil levels and TAC values for asthma, COPD, and ACO patients, no significant relationship was found (*p* = 0.548, *p* = 0.537, *p* = 0.907, respectively) (Table 3). Also, the mean neutrophil count was 3460.93 ± 1444.51, and the mean lymphocyte count was 2036.80 ± 731.11. There was no statistically significant difference between neutrophil and lymphocyte counts and TAC level (*p* = 0.456 and *p* = 0.445, respectively).

## 4. Discussion

In this cross-sectional study, 76 patients diagnosed with asthma, COPD, or ACO were evaluated in terms of their TAC levels. The TAC levels were lower in COPD patients than in asthma and ACO patients. Our results show that TAC levels were lower in current and former smokers compared to never smokers. Our study revealed no significant correlation between TAC level and eosinophil count and FEV1 or FEV1/FVC. TAC level may be used as a marker to differentiate COPD from asthma or ACO.

In a study evaluating the total antioxidant status of patients with COPD during exacerbation and after stabilization, it was found to be significantly lower in both conditions compared to the control group [17]. Comparing the results of Japanese patients with asthma, COPD, and ACO, the glutathione level (an antioxidant) was particularly low in patients with COPD [18]. Similarly, a study conducted in our country observed a significant increase in oxidative stress in COPD patients, but no increase in antioxidant response was found [19]. In a study of 60 COPD patients, total serum antioxidant levels were significantly lower in COPD patients and healthy smokers compared to healthy non-smokers [20]. Although there were no healthy controls in our study, it has been shown that having COPD leads to lower TAC levels, even when compared with two other inflammatory diseases.

In addition, TAC levels were found to be higher in never smokers than in former and current smokers according to our results. However, since there were no never-smokers in the COPD group in our study, it could not be compared. Cigarette smoke is one of the biggest confounders in antioxidant evaluation in patients with COPD, as it causes oxidative stress because of ROS activation and decreases antioxidant levels. In one study, antioxidant levels were found to be similar between former and current smokers with COPD. Antioxidant levels were found to be significantly lower in COPD patients who were current smokers compared with non-COPD individuals who were current smokers. These results also prove that smoking is not the only factor [21].

In a study that divided COPD into four groups according to disease severity, TAC levels were found to be lowest in the most severe group. In the same study, a moderate correlation was found between FEV1 and TAC levels [22]. Consistent with our study, no significant difference was found between the severity of airway obstruction and total antioxidant levels in COPD patients in the study of Hlavati et al. [23]. Similar to our study, no significant correlation was found between total antioxidant status and pulmonary functional parameters in a study including patients with COPD who were not in an attack state [24]. The different results between the degree of obstruction and TAC levels were similar for asthma. Serum TAC levels correlated with FEV1 in 104 asthma patients in a Korean study (r = 0.22, *p* = 0.03). It was found that the group with higher baseline TAC levels maintained a higher mean FEV1 value both 1 and 2 years after enrolment [25]. In our study, no correlation was found between TAC levels and FEV1 levels in the asthma group. This may be because the number of asthma patients in our study was lower than in the mentioned study, and follow-up spirometry values of our patients were not taken.

In another study, malondialdehyde and protein carbonyl levels, which are markers of oxidative stress, were higher in the uncontrolled group compared to fully controlled and partially controlled asthmatic patients. Glutathione levels, an antioxidant, decreased in all subgroups. Therefore, oxidative and antioxidative markers can be used to reflect asthma control [26]. In another study of total oxidant and antioxidant levels in asthmatic patients compared with a control group, no statistically significant difference was found between the two groups. Although inflammation was high in asthmatic patients, the authors stated that these results were because the treatment applied prevented excessive oxidant formation [27]. Similarly, total antioxidant levels were compared in patients with stable and acute asthma, and total antioxidant levels were found to be inversely related to the severity of asthma [28]. As far as we have found, there have been no studies investigating eosinophil counts and TAC levels among these three disease groups. It is estimated that eosinophils also show inflammation and exacerbations in asthma patients through oxidative damage. However, in our study, no significant relationship was found between eosinophil counts, which play an important role in type 2 inflammation in asthma, and TAC levels among the three groups. The reason for the lack of difference in our study may be that clinically stable asthmatic patients were not included in this study.

In a study comparing total antioxidant activity at diagnosis and 3 months after treatment in patients treated for asthma, it was shown that there was a decrease in total antioxidant activity in both saliva and serum with treatment. Therefore, another perspective is that total antioxidant levels are a defense mechanism related to the severity of the disease. It is not fully understood whether low antioxidant levels cause obstructive lung diseases or whether antioxidant levels increase in proportion to the defense and severity of these diseases. This uncertainty can be prevented by studies comparing the stable and exacerbation periods of the disease and determining the threshold values of the parameters in which antioxidant levels are studied compared to healthy individuals, because it can be seen that the ideal antioxidant level for the absence of obstructive lung disease has not yet been determined.

Our study has some limitations. The exclusion criteria could have been widened, especially in terms of diseases that may affect oxidant/antioxidant activity. Patients could have been selected more homogenously in terms of age and gender. Other limitations include the fact that the effect of diet and drug treatment on TAC levels could not be included in the analysis. Also, the study did not conduct a formal cost analysis. However, we know that TAC tests have the potential to be a relatively inexpensive, minimally invasive, and easily administered adjunctive tool in many laboratory settings. The potential value of conducting cost–benefit analyses in the future is therefore important. Additionally, we can emphasize that larger, multicenter studies will enhance the generalizability of our findings. On the other hand, since there are not enough studies comparing asthma, COPD, and ACO groups, we believe that our study will shed light on this intriguing issue. Furthermore, our findings could be an important step towards future studies that could develop diagnostic thresholds or composite indices incorporating TAC.

## 5. Conclusions

COPD is a disease in which oxidative stress is present at all stages, in terms of smoking, environmental, and occupational exposure, being a systemic inflammatory disease, its comorbidities, and the course of the disease. Oxidative stress increases and antioxidant capacity decreases in smoking patients with COPD. TAC levels are a marker that can be used in patients with a pre-diagnosis of ACO, especially in differentiating from COPD. Regarding these results, the demonstration of decreased antioxidant activity in patients with COPD will guide future treatment strategies.

## Figures and Tables

**Table 1:** Demographic data of the patients.

Variable	n (%)
Gender	
Male	47 (61.8)
Female	29 (38.2)
Smoking status	
Never smoker	15 (19.7)
Former smoker	22 (28.9)
Current smoker	39 (51.3)
Diagnosis	
Asthma	26 (%34.2)
COPD	30 (%39.5)
ACO	20 (%26.3)
	Mean ± SD
Age	55.6 ± 14.4
Spirometry	
FEV1%	69.9 ± 21.1
FVC%	73.4 ± 19.3
FEV1/FVC	73.3 ± 11.8
Total antioxidant level (mmol/L)	1079.1 ± 261.4

SD: standard deviation; COPD: chronic obstructive pulmonary disease; ACO: asthma COPD overlap.

**Table 2:** Comparison of TAC levels of various groups *.

	TAC Level (mmol/L) Mean ± SD	*p* Value
Diagnosis		
Asthma	1154.8 ± 310.1 ^b^	0.192
COPD	1027.9 ± 203.6 ^a^	
ACO	1042.4 ± 260.9 ^b^	
Gender		
Male	1035.6 ± 245.5	0.065
Female	1149.6 ± 275.2	
Smoking status		
Never smoker	1167.9 ± 254.8 ^a^	0.960
Former smoker	1091.7 ± 320.9 ^b^	
Current smoker	1076.3 ± 261.4 ^b^	

* Independent-sample *t*-test, one-way ANOVA. SD: standard deviation; COPD: chronic obstructive pulmonary disease; ACO: asthma COPD overlap; TAC: total antioxidant capacity. In post hoc analyses, the same letter indicates no statistically significant difference, while different letters indicate a statistically significant difference.

**Table 3:** The relationship between eosinophil count, FEV-1, FEV1/FVC, and TAC level according to diagnosis *.

	Within-Group Correlations for TAC Levels					
	Eozinofil (rho_S_)	*p* Value	FEV1 (rho_S_)	*p* Value	FEV1/FVC (rho_S_)	*p* Value
Asthma	−0.124	0.548	−0.351	0.079	−0.001	0.995
COPD	−0.117	0.537	−0.282	0.132	−0.003	0.986
ACO	0.028	0.907	−0.065	0.784	0.171	0.471

COPD: chronic obstructive pulmonary disease; ACO: asthma–COPD overlap; TAC: total antioxidant capacity. rho_S_: Spearman correlation coefficient. *: Spearman’s correlation test.

## Data Availability

The data of this study can be requested from the corresponding author when certain acceptance reasons are presented.

## References

[1] Rogers L.K., Cismowski M.J. (2018). Oxidative stress in the lung—The essential paradox. Curr. Opin. Toxicol..

[2] Barnes P.J., Shapiro S.D., Pauwels R.A. (2003). Chronic obstructive pulmonary disease: Molecular and cellular mechanisms. Eur. Respir. J..

[3] Thimmulappa R.K., Chattopadhyay I., Rajasekaran S. (2019). Oxidative stress mechanisms in the pathogenesis of environmental lung diseases. Oxidative Stress in Lung Diseases.

[4] Global Initiative for Chronic Obstructive Lung Disease (2024). Global Strategy for the Diagnosis, Management, and Prevention of COPD. https://goldcopd.org.

[5] Barnes P.J. (2022). Oxidative stress in chronic obstructive pulmonary disease. Antioxidants.

[6] Kume H., Yamada R., Sato Y., Togawa R. (2023). Airway smooth muscle regulated by oxidative stress in COPD. Antioxidants.

[7] Global Initiative for Asthma (2024). Global Strategy for Asthma Management and Prevention. https://ginasthma.org.

[8] Kleniewska P., Pawliczak R. (2017). The participation of oxidative stress in the pathogenesis of bronchial asthma. Biomed. Pharmacother..

[9] Bezerra S., Maretto B.T., Felix S.N., Cirillo J.V.O., Araújo K.A., Moreira A.R., Ayres S.A., Laia R.M., Cruz F.M., Almeida F.M. (2024). Evaluation of inflammation, remodeling, hyperresponsiveness and oxidative stress in experimental models of asthma–COPD overlap. Eur. Respir. J..

[10] Mohideen K., Chandrasekaran K., Veeraraghavan H., Faizee S.H., Dhungel S., Ghosh S. (2023). Meta-analysis of assessment of total oxidative stress and total antioxidant capacity in patients with periodontitis. Dis. Markers.

[11] Silvestrini A., Meucci E., Ricerca B.M., Mancini A. (2023). Total antioxidant capacity: Biochemical aspects and clinical significance. Int. J. Mol. Sci..

[12] Global Initiative for Asthma (2018). Global Strategy for Asthma Management and Prevention. https://ginasthma.org.

[13] Global Initiative for Chronic Obstructive Lung Disease (2018). Global Strategy for the Diagnosis, Management, and Prevention of COPD. https://goldcopd.org.

[14] Centers for Disease Control and Prevention (CDC) Smoking Glossary. https://archive.cdc.gov/#/details?url=https://www.cdc.gov/nchs/nhis/tobacco/tobacco_glossary.htm.

[15] Erel O. (2004). A novel automated direct measurement method for total antioxidant capacity using a new generation, more stable ABTS radical cation. Clin. Biochem..

[16] Re R., Pellegrini N., Proteggente A., Pannala A., Yang M., Rice-Evans C. (1999). Antioxidant activity applying an improved ABTS radical cation decolorization assay. Free Radic. Biol. Med..

[17] Stanojkovic I., Kotur-Stevuljevic J., Milenkovic B., Spasic S., Vujic T., Stefanovic A., Llic A., Ivanisevic J. (2011). Pulmonary function, oxidative stress and inflammatory markers in severe COPD exacerbation. Respir. Med..

[18] Kodama Y., Kishimoto Y., Muramatsu Y., Tatebe J., Yamamoto Y., Hirota N., Itoigawa Y., Atsuta R., Koike K., Sato T. (2017). Antioxidant nutrients in plasma of Japanese patients with COPD, asthma–COPD overlap syndrome and bronchial asthma. Clin. Respir. J..

[19] Cansiz K., Tug T., Konuk S. (2020). Oxidant and antioxidant balance in stable COPD patients and during acute COPD exacerbations. Med. Sci..

[20] Salama R.H.M., Elkholy M.M., Sadek S.H., Mahdy I.G. (2017). Total antioxidant capacity as a marker in predicting severity of COPD. Egypt. J. Bronchol..

[21] Aydemir Y., Aydemir Ö., Şengül A., Güngen A.C., Çoban H., Taşdemir C., Düzenli H., Şehitoğulları A. (2019). Comparison of oxidant/antioxidant balance in COPD and non-COPD smokers. Heart Lung.

[22] Ahmad A., Shameem M., Husain Q. (2013). Altered oxidant-antioxidant levels in the disease prognosis of COPD. Int. J. Tuberc. Lung Dis..

[23] Hlavati M., Tomić S., Buljan K., Buljanović V., Feldi I., Butković-Soldo S. (2020). Total antioxidant status in stable chronic obstructive pulmonary disease. Int. J. Chronic Obstr. Pulm. Dis..

[24] Bekir S. (2022). Association between oxidative stress markers and lung function parameters in stable COPD patients. Turk. J. Vasc. Surg..

[25] Yoon S.Y., Kim T.B., Baek S., Kim S., Kwon H.S., Lee Y.S., Lee T., Jang A.S., Chang Y.S., Cho S.H. (2012). The impact of total antioxidant capacity on pulmonary function in asthma patients. Int. J. Tuberc. Lung Dis..

[26] Karadogan B., Beyaz S., Gelincik A., Buyukozturk S., Arda N. (2022). Evaluation of oxidative stress biomarkers and antioxidant parameters in allergic asthma patients with different level of asthma control. J. Asthma.

[27] Emecen Ö., İnal B.B., Erdenen F., Usta M., Aral H., Güvenen G. (2010). Evaluation of oxidant/antioxidant status and ECP levels in asthma. Turk. J. Med. Sci..

[28] Fatani S.H. (2014). Biomarkers of oxidative stress in acute and chronic bronchial asthma. J. Asthma.

